# Assessment of dietary patterns and macronutrient intake among the adult population of Armenia

**DOI:** 10.3389/fnut.2026.1840868

**Published:** 2026-05-20

**Authors:** Davit Pipoyan, Meline Beglaryan, Bagrat Harutyunyan, Sara Monteiro Pires

**Affiliations:** 1 Center for Ecological-Noosphere Studies (CENS), National Academy of Sciences of the Republic of Armenia (NAS RA), Yerevan, Armenia; 2 National Food Institute, Technical University of Denmark (DTU), Kongens Lyngby, Denmark

**Keywords:** diet, food consumption, macronutrient, nutrient intake, nutrition

## Abstract

**Background:**

Inadequate dietary intake is one of the crucial contributors to the growing burden of diet-related non-communicable diseases. However, in South Caucasus countries including Armenia, limited evidence exists on individual-level nutrient intake, challenging efforts toward effective nutrition interventions. This study is the first attempt in Armenia to assess dietary habits and macronutrient intakes using an individual-based approach.

**Methods:**

The Armenian food composition table by FAO was used to estimate energy and nutrient intake. The analysis covered 124 food items and 40 mixed dishes. Food consumption data were sourced from a nationally representative, individually based database available in Armenia (*n* = 1400 adult residents). This database provides detailed information on actual food intake, including food preparation methods and foods consumed outside the home. Data analyses were performed using Anaconda’s Jupyter Notebook and SPSS Software.

**Results:**

Dietary patterns were dominated by bread and flour-based products, which represented the main source of energy and macronutrients in the studied population’s diet. The mean total energy intake per day was 1738 kcal. Mean daily intakes of protein, carbohydrates, and total fat were 71.51 g/day, 209.57 g/day, and 69.91 g/day, respectively. These values fall below the recommended levels established by the Eurasian Economic Union (EAEU). Compared to the Dietary Reference Values (DRVs) set by the European Food Safety Authority (EFSA), only a small proportion of respondents met the reference intake ranges for carbohydrates and fats, while 70% met the average requirement for protein.

**Conclusion:**

The findings indicate a dietary imbalance and strong reliance on flour-based products, highlighting the need for health policies and interventions to improve dietary diversity in Armenia and the broader Caucasus region.

## 1 Introduction

Diet, nutritional status, and physical activity are recognized as major determinants of health particularly of non-communicable diseases (NCDs) (1). In the past decades, these determinants have undergone dramatic changes affecting food consumption patterns, food security, and environmental sustainability. As the society transitioned from an agricultural economy to a manufacturing economy and an information-gathering sedentary society, energy intake dropped by more than half (2). Besides the changing modern lifestyle, nowadays, as the consumption of processed and convenience foods is rising, more calories are obtained from meat, sugars, oils, and fats than from fiber-rich foods such as whole grains, pulses, and roots (3, 4). This, in turn, leads to changes in overall energy and macronutrient intake, resulting in a deficiency or excess of these substances (5–7). Macronutrients, including protein, carbohydrates, and fats play a vital role in ensuring a healthy diet and preventing many diseases. They can be beneficial as well as harmful depending on the amount received (8).

In Armenia, limited literature is available exploring the nutritional status and energy intake. Several studies highlight the fact that although people can achieve the daily recommended calorie intake, the nutrient requirements might not be met, leading to malnutrition and long-term health implications (9, 10). The population has long been characterized by nutrient deficiencies, including vitamin A deficiency or iron deficiency anemia (11–13). Moreover, the adult Armenian population’s daily diet is mainly based on foods of plant origin, with the predominance of carbohydrate sources such as bread and related products. The reasons for poor diets are diverse including food accessibility constraints, such as insufficient resources to purchase nutrient-rich products, and educational constraints, such as lack of proper nutritional education and knowledge about nutritious foods (13, 14).

A recent study conducted by the Informational-Analytical Center for Risk Assessment of Food Chain used a household budget survey to explore food consumption trends and assess major macronutrient intakes over the last decade in Armenia (15). Similar to the findings of other studies, it showed that approximately more than 50% of total energy, protein, and carbohydrate intake is attributable to cereals and bakery products. Over time, the energy and macronutrient intakes of Armenians have decreased, while the contribution of each food group to total energy and nutrient intake has not changed. Furthermore, the macronutrient intake is not balanced among the adult population. In particular, the amounts of energy and carbohydrate intake are below the recommended values set by the World Health Organization (WHO) and Food Agriculture Organization (FAO), total fat intake is at the highest recommended level, while the amount of protein exceeds the threshold (15).

A major drawback of these studies, however, is that they do not focus on the macronutrient intake trends on an individual level since the studies are conducted via household surveys. Despite their advantages, household-based surveys are characterized by many shortcomings. These surveys aim to evaluate the financial and living conditions of households, rather than specific nutrition and food consumption habits. Other disadvantages include the short recall reference periods and the insufficient accounting for foods that are wasted, kept for storage, or consumed away from home. Also, household surveys do not take into consideration the effects of cooking on food weight and nutrient content (16, 17). Due to the absence of data on distributions of intakes within household members according to individual characteristics such as age, gender, education, or income, the validity of HCES for estimating individual dietary intake is not well established (16). In contrast to household-based surveys, individual-based surveys, such as 24-h recall (24HR) or Food Frequency Questionnaire (FFQ) are considered better tools for nutritional assessment. For example, 24HR can capture the actual food intake at the individual level. It can also account for food preparation methods and food consumed away from home (18).

Therefore, there is a need to investigate dietary habits among Armenian households, especially on an individual level to get more accurate data based on age, gender, and other characteristics. It is important to note that, currently, there is an absence of an official individual-based methodology for investigating dietary habits among Armenian households, thus making it difficult for policymakers to monitor changes using evidence-based approaches. The study aims to address this gap by conducting the first individual-level assessment of dietary habits and macronutrient intakes in Armenia.

## 2 Materials and methods

### 2.1 Data sources and food classification

This study used a nationally representative, individually based dietary intake database available in Armenia, including 1,400 adult respondents aged 18–80 years (19). The dataset was derived from an anonymous quantitative 24-h dietary recall (24HR) survey, which recorded all foods consumed by each respondent during the previous day (19). The database comprised 164 items, including 124 individual food items and 40 mixed dishes. For dietary estimation purposes, all food items were classified into 11 major food groups (bread and flour-based products, milk and milk products, meat and meat products, fat and oil products, fish, eggs, fruits, vegetables, potatoes, sugar, honey and confectionery, juices) and 4 mixed dish groups (salads, soups, pilafs (with rice, buckwheat, emmer, etc.), and scrambled eggs). A complete list of all food items and their grouping is provided in Supplementary Table S1.

Mixed dishes (salads, soups, pilafs and scrambled eggs) were treated as composite food items and were not disaggregated into individual ingredients, as they were reported and consumed in their final prepared form.

Nutrient composition data were obtained from the Armenian food composition table, developed jointly by the National Statistical Service of the Republic of Armenia, the Ministry of Agriculture, and the Food and Agricultural Organization (FAO) of the United Nations. The food composition table was compiled in accordance with INFOODS standards and food components identifiers (20).

Each food item in the consumption database was matched to a corresponding entry in the food composition table to obtain nutrient values per 100 g of food.

### 2.2 Assessment of macronutrient and energy daily intake

Daily intake of macronutrients (protein, carbohydrate, and total fats) and total energy was calculated for each respondent of the 24HR survey based on reported food consumption. As the 24HR method captures foods in ready-to-eat form, all estimations were conducted on an “as consumed” basis.

Individual macronutrient intake was calculated by multiplying the amount of each consumed food item by its corresponding macronutrient content and summing across all consumed foods, according to the Equation (1) (15):

$$I=\sum_{i=1}^{n}(W_{i}\times C_{i})$$ (1)

Where *I* is the total daily intake of a given macronutrient (g/day), *W_i_* is the amount of *i* food item consumed (g/day), *C_i_* is the macronutrient content of *i* food item (g/100 g), and *n* is the total number of consumed food items.

Total daily macronutrient intake was obtained by summing contributions from all food groups.

Total metabolizable energy intake was calculated using standard conversion factors, i.e., 4 kcal/g for protein, 4 kcal/g for carbohydrates, and 9 kcal/g for total fats.

### 2.3 Statistical analysis of data

The statistical analyses were performed using IBM SPSS Software (SPSS Inc., Chicago, Ill., USA, version 28) as well as Anaconda’s Jupyter Notebook. Five Python packages, including Pandas, NumPy, Seaborn, Scikit-learn, and Matplotlib were applied.

Food consumption data was analyzed using normality tests to check the homogeneity of values. According to the significance value of the Shapiro–Wilk Test (*p* < 0.05), the consumption data significantly deviates from a normal distribution. Outliers were identified using boxplots. The interquartile range (IQR) method was applied to remove them since it is the most appropriate method for skewed data.

Differences in macronutrient and energy intakes between gender groups were evaluated. Due to a nonparametric distribution, the Mann–Whitney U test and Kruskal-Wallis analysis of variance (ANOVA) were used to compare intake between gender groups, with a *p*-value of less than or equal to 0.05 as a level of significance. A Mann–Whitney U test was used to compare intake between two groups of interest: males and females.

## 3 Results and discussion

### 3.1 Dietary patterns

**Figure 1:**
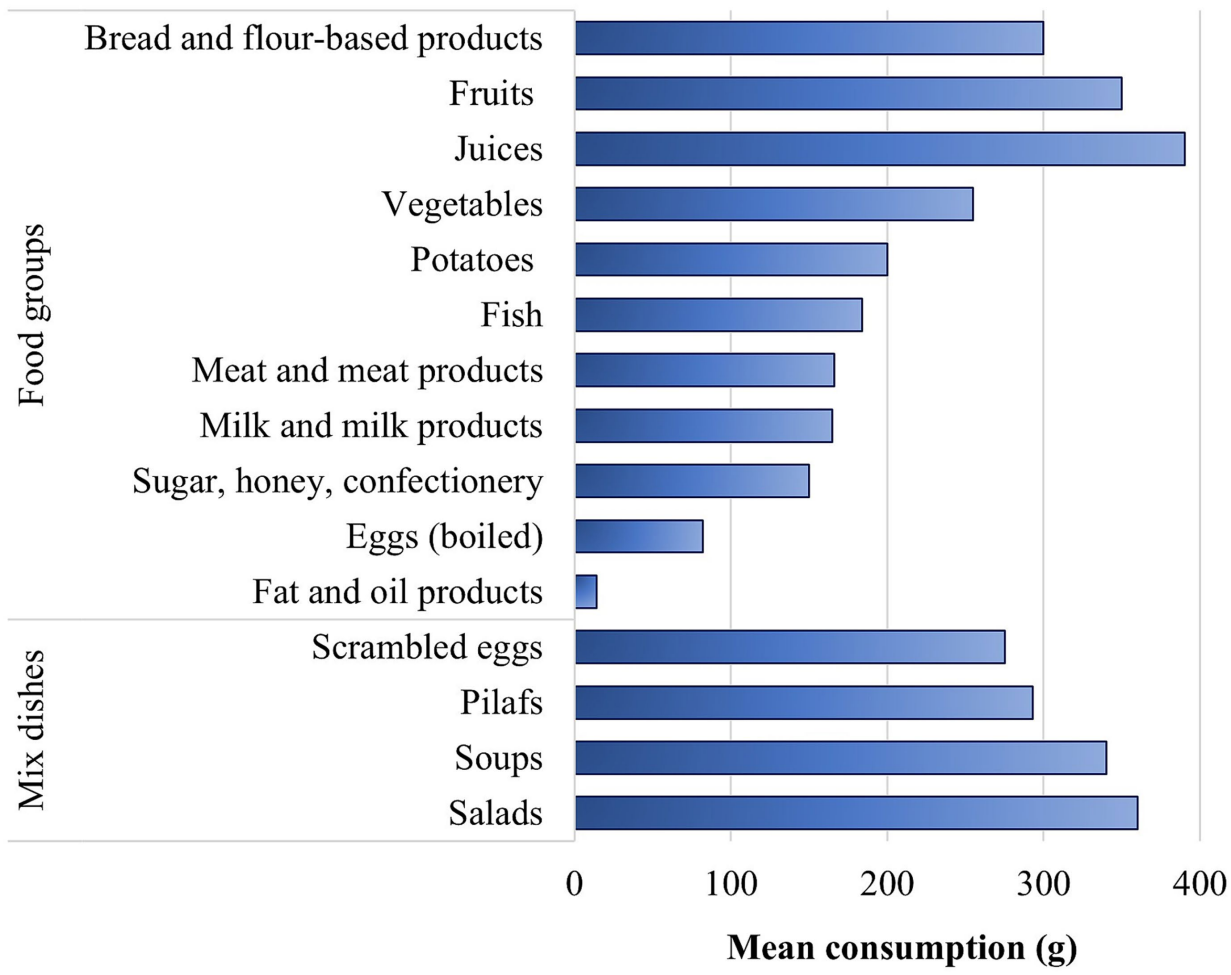
Mean consumption (g) of main food groups and mixed dishes during the past 24 h.

Food consumption data from the 24HR survey reflect individual food intake during the past 24 h for the total study population (*n* = 1,400) (19). While a single 24HR does not capture intra-individual day-to-day variability in dietary intake and may be subject to recall bias, it provides detailed quantitative information on actual food consumption at the individual level. Importantly this is the first study in Armenian to investigate dietary patterns using nationally representative individual-level 24HR data, allowing a more accurate estimation of food group consumption compared with previous household-based approaches (15). Figure 1 shows the mean consumption across 11 food groups (bread and flour-based products, milk and milk products, meat and meat products, fat and oil products, fish, eggs, fruits, vegetables, potatoes, sugar, honey and confectionery, juices) and 4 mixed dish groups. Mixed dishes constituted separate food groups in the dataset and included salads, soups, pilafs, and scrambled eggs. Intake amounts (g/day) refer to consumers only (i.e., 24HR survey respondents who reported consumption of a given food item).

In this study, bread and flour-based products were the most widely consumed food group, reported by approximately 96% of the studied population, with an average intake of 300 g. The proportion of the adult population consuming first-grade wheat bread is 2.5 times higher than that of those consuming ‘Lavash’, a thin flatbread (21). Similarly, the average intake for first-grade wheat bread (175 g) is 2.5 times higher than that of ‘Lavash’ (70 g). A major part of flour-based product consumption is attributable to the intake of biscuits, cakes, pastries, waffles, and cookies, with 40% of the studied population consuming these foods. Pasta (such as spaghetti, and vermicelli) is consumed by approximately 13% of the studied population, with an average intake of 250 g. Approximately 20% of the studied population consumes other cereals, including groat, oat, buckwheat, and rice, with a mean intake of 155 g. Rice dishes, such as lentils with rice and rice with chicken meat are consumed by 2% of the studied population, with a mean intake of 293 g.

Milk and milk products (including pasteurized cow milk, condensed milk, cheese, yogurt, kefir, matsun, tan, sour cream, and curd cheese) are consumed by 70% of the studied population, with an average intake of 165 g. Among these, cheese is consumed by almost half of the studied population. Matsun and sour cream are consumed by 17 and 8% of studied population, respectively. The other milk products are consumed by a small number of people.

Meat and meat products are consumed by 55% of the studied population and meat-based dishes are consumed by approximately 17% of them. The average intake of meat and meat products among consumers (i.e., 24HR survey respondents who reported consumption of a given food) is approximately 166 g. Chicken meat is the most commonly consumed meat (18% of the studied population) followed by sausages (14% of the studied population) and beef and veal (13% of the studied population). Fish is not a typical food in the Armenian diet as it is usually consumed only during certain occasions (22). In this 24-h recall study, it is consumed by 2% of the studied population with an average intake of approximately 184 g.

Eggs, particularly chicken eggs are widely consumed among Armenians. In this research, 11% of the studied population consumed boiled eggs and 18% consumed scrambled eggs in the form of a mixed dish. The mean intake values of boiled and scrambled eggs amount to 82 g and 275 g, respectively. The following types of scrambled eggs are the most popular according to this study: scrambled eggs with tomato, sausages, cheese, green beans, and greens.

Fat and oil products, such as butter, margarine, and spread are consumed by as much as 11% of the studied population with an average intake of 14 g.

Fruits and vegetables are consumed by the majority of the respondents (47 and 55%, respectively). The average combined intake of fruits and vegetables (excluding watermelon and melon) during the past 24 h is approximately 328 g. The WHO recommends a daily intake of 400 g of fruit and vegetables (23). In this study, approximately 70% of those who consumed fruits and vegetables (excluding watermelon and melon) during the past 24 h, receive less than the recommended value. The consumption of watermelon and melon is characterized by seasonal variation as well as the big weight of these foods. Therefore, when considering the consumption of these foods too, the average combined intake of fruits and vegetables in the past 24 h amounts to 400 g, with 63% of consumers (i.e., 24HR survey respondents who reported consumption of a given food) receiving less than the recommended value set by WHO. Potatoes are consumed by 31% of the studied population with an average intake of approximately 200 g. Potatoes are an important part of the Armenian diet and are consumed in various ways, including fried, boiled, smashed, oven-baked, or French fried.

Confectionery including sugar, honey, chocolate, and sweet snacks is widely consumed among the Armenian population, with 73% of respondents consuming these food items. The average intake amounts to 150 g. In particular, granulated sugar and chocolate are each consumed by 31% of the studied population. The mean intakes of granulated sugar and chocolate are 11 g and 26 g, respectively. Another big portion is attributable to ice cream with 20% of people consuming it in the past 24 h with a mean intake of 110 g.

Among the mix dishes considered in this study, salads are consumed by approximately 28% of the respondents, with an average intake of 360 g. The most widely consumed salads are ‘Summer salad’ and ‘Cabbage salad’. Soups and other meat-containing traditional dishes are consumed by 20% of the studied population, with an average intake of 340 g. ‘Soup with chicken meat’, ‘Soup with beef’, ‘Bortsch’, and ‘Spas’ are consumed the most. Soups such as ‘Bortsch’, and ‘Spas’ are very common among former Soviet Union countries (24).

Overall, the dietary pattern of the studied population is characterized by a high reliance on staple foods, particularly bread and flour-based products (particularly bread), potatoes, and dairy products, which are consumed by the majority of the studied population regardless of age and gender. National statistics further support the observed dietary pattern. According to the Food Security and Poverty reports (January–December 2025) of the Statistical Committee of Armenia, the national average proportion of the whose diet is composed of 70% bread and potatoes was 10.5% in 2023 and 5.3% in 2024. Despite this overall decrease, regional disparities remain, with higher proportions reported in certain regions, reaching 30.1% in 2023 and 19.7% in 2024 (25). These data indicate heterogeneity in dietary dependence on staple foods across the country while remaining consistent with the overall dietary pattern observed in this study.

### 3.2 Macronutrient and energy intake

**Figure 2:**
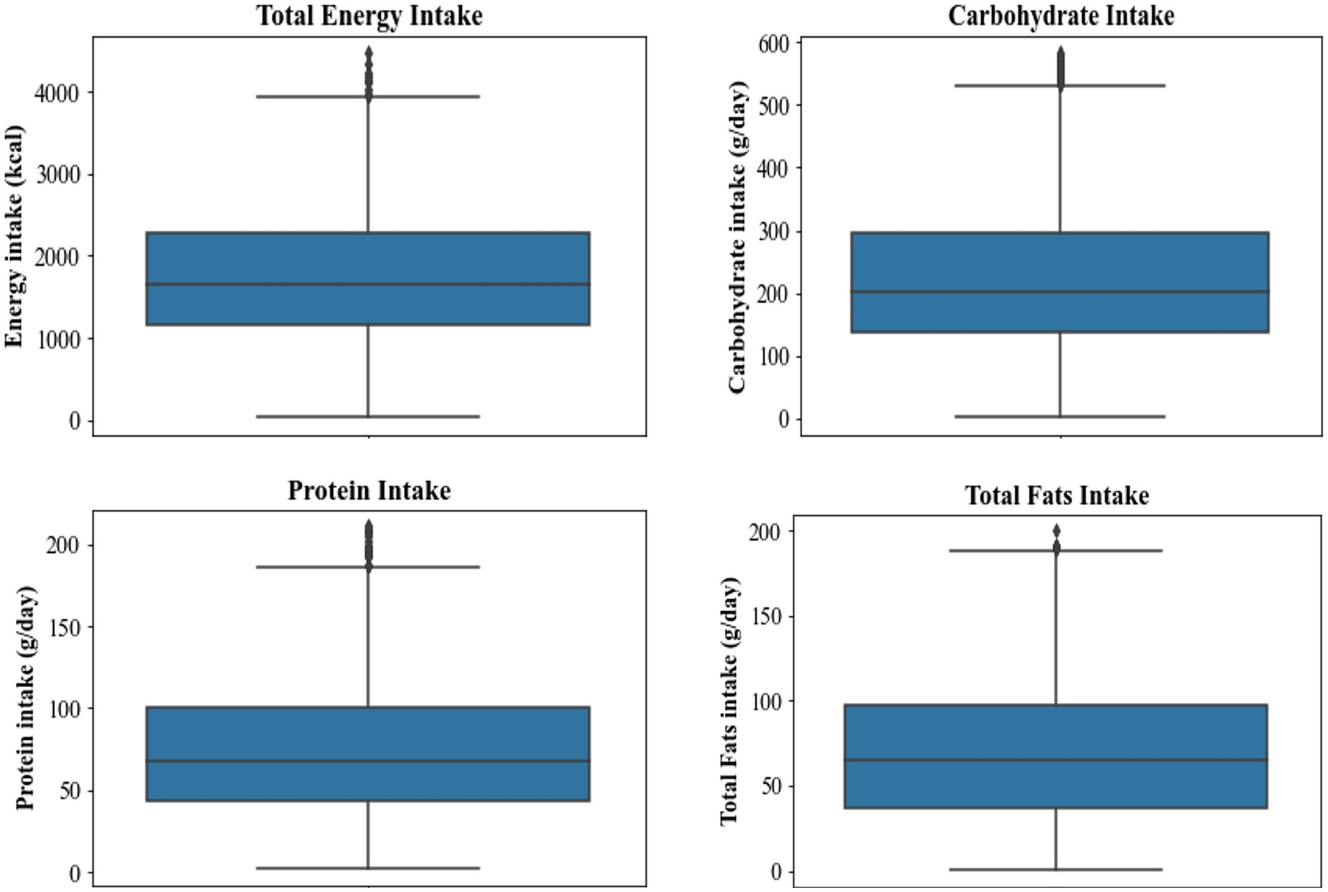
Estimated intakes of total energy and macronutrients during the past 24 h.

The mean estimates of protein, carbohydrates, and total fats are equal to 71.51 g/day, 209.57 g/day, and 69.91 g/day, respectively (Figure 2). The 75th percentile receives less than 95 g of protein, 274 g of carbohydrate, and 97 g of total fats, the other 25th percentile of the studied population receives more than these amounts. Currently, Armenia does not have its Dietary Reference Values (DRVs) for the studied indicators. However, Armenia is a member of the Eurasian Economic Union (EAEU) and follows its Customs Union Technical regulations for food product labeling (26). The latter defines the average daily need for basic nutrient substances and energy. Based on EAEU technical regulation on food products in terms of their labeling, the average daily requirement for basic nutrients, including protein, carbohydrates, and total fats, for labeling food products should be equal to 75 g, 365 g, and 83 g, respectively (26). Thus, the estimated average intakes of all macronutrients are below the EAEU recommended values.

European Food Safety Authority (EFSA) provides Dietary Reference Values (DRVs), such as Average Requirement (AR), Reference Intake (RI) ranges, and Population Reference Intake (PRI) to indicate the amount of a nutrient that must be consumed regularly to maintain health for a healthy individual (27). Regarding proteins, the EFSA Panel considers that the value of 0.66 g/kg body weight per day can be accepted as the AR and the value of 0.83 g/kg body weight per day as the PRI (27). In this study, the estimated mean protein intake is equal to approximately 1.01 g/kg body weight, which is higher than the AR and PRI set by the EFSA. However, when looking at the 75th percentile, the majority of the respondents receive less than 0.83 g/kg body weight of protein. Given as a percentage of total energy intake (E%), EFSA proposes 45 to 60 E% as the Reference Intake range for carbohydrates. Diets with glycemic carbohydrate contents of 45 to 60 E%, in combination with reduced intakes of fat and saturated fatty acids (SFA), are compatible with the improvement of metabolic risk factors for chronic disease (27). Compared to this range, the estimated mean metabolized energy received from carbohydrates (48 E%) falls within this range. Moreover, the estimated metabolized energy for the 75th percentile equals 63.1 E%. Regarding total fats, the EFSA Panel sets a lower bound of the Reference Intake range of 20 E% and an upper bound of 35 E% for adults (27). Compared to this range, the estimated mean metabolized energy received from total fats (36.2 E%) exceeds the threshold. It should be noted that the 75^th^ percentile is equal to 50.5 E%, which is quite higher than the threshold. Overall, compared to the DRVs set by EFSA, only 21% of respondents meet the RI range for carbohydrates, 70% meet the AR for protein, and 25.3% meet the RI range for total fats.

The mean amount of total energy estimated from the consumption of foods during the past 24 h amounts to 1738 kcal (Figure 2). The 75th percentile of the studied population receives less than 2,270 kcal total energy, while the other 25th percentile receives more than that. Based on EAEU regulation (26), the required daily intake of energy should be equal to 2,500 kcal. Thus, the estimated average amount of energy intake is below the recommended value.

EFSA established ARs of energy based on age groups (except for adults ≥ 80 years) and physical activity level (PAL). Considering a PAL of 1.4 to approximately reflect a low active (sedentary) lifestyle, the estimated mean energy values for almost all age and gender groups are below the AR (Table 1). Meanwhile, approximately 25–30% of the respondents meet the AR.

**Table 1:** Estimated energy intake (kcal/day) compared with the AR set by EFSA.

Age groups	Mean energy intake (kcal/day)		75th percentile energy intake (kcal/day)		AR for energy (kcal/d) at Physical activity level = 1.4	
	Male	Female	Male	Female	Male	Female
18–29	1962.3	1809.3	2439.0	2310.9	2340.5	1886.8
30–39	1892.2	1553.6	2432.2	1987.1	2268.9	1815.1
40–49	1993.7	1542.1	2609.6	2033.8	2221.1	1791.2
50–59	1927.3	1561.2	2410.0	2015.8	2197.2	1791.2
60–69	1851.8	1645.1	2372.7	2134.0	2006.2	1624.0
70–79	1558.6	1509.9	2004.9	1670.2	1982.3	1624.0

When comparing total energy estimated values with the average energy requirements set by the WHO and FAO (1650–3,150 kcal/day for women, and 2,100–3,600 kcal/day for men depending on physical activity level), the mean estimated energy intakes are less than the requirement both for women and men (1,608 kcal for women and 1892 kcal for men) (28). Only 25% of the studied population meets the average energy requirement; 25% of women receive more than 2050 kcal/day and 25% of men receive more than 2,415 kcal/day. A Mann–Whitney U test was used to compare the energy intake between two groups of interest: males and females. It was revealed that energy intake is statistically different between these gender groups (*p* < 0.05). Thus, men receive approximately 284 kcal more than women. The observed difference may be attributed to gender-related variations in dietary patterns, particularly differences in both the types and amounts of foods consumed, including a higher consumption of energy-dense food products among men. Overall, this reflects distinct food consumption behavior within the studied population. When comparing the Armenian diet with the diets of neighbouring developing countries as well as with developed countries, noticeable differences are observed. In the Armenian diet, the mean intake of energy (1738 kcal) is lower compared with that of developed countries, such as Australia (2030 kcal), the United States (2076 kcal), France (2162 kcal), Denmark (2125 kcal), and Netherlands (2273 kcal) (29).

The results of the current study are largely similar to a 2022 investigation conducted by the Informational-Analytical Center for Risk Assessment of Food Chain at the Center for Ecological-Noosphere Studies of RA, aimed to investigate the trends in food consumption and nutrient intakes based on household budget survey. In both studies, the estimated average intakes of all macronutrients (except for protein) and total energy are below the EAEU recommended values. In the case of the current 24HR study, the mean protein intake is below the EAEU threshold. Similarly, in both studies, mean metabolized energy amounts received from carbohydrates fall within the recommended range set by EFSA. Mean metabolized energy amounts received from total fats and protein exceed the respective thresholds set by EFSA (15). However, from a methodological perspective, it should be emphasized that the current study is based on a single 24HR, which is a major study limitation as according to multiple studies, energy intake is underreported in the first 24HR due to the number of food omissions. While this approach provides detailed quantitative data at the individual level, it may not fully represent habitual intake, and may therefore contribute to uncertainty in estimating usual nutrient intake distribution. Three 24HRs appear optimal for estimating energy intake (30, 31). In general, repeated 24HR data collected on two non-consecutive days, together with statistical modelling approaches, can be used to estimate the distribution of usual intake among the population (32).

### 3.3 Food sources of macronutrient intake and energy

**Figure 3:**
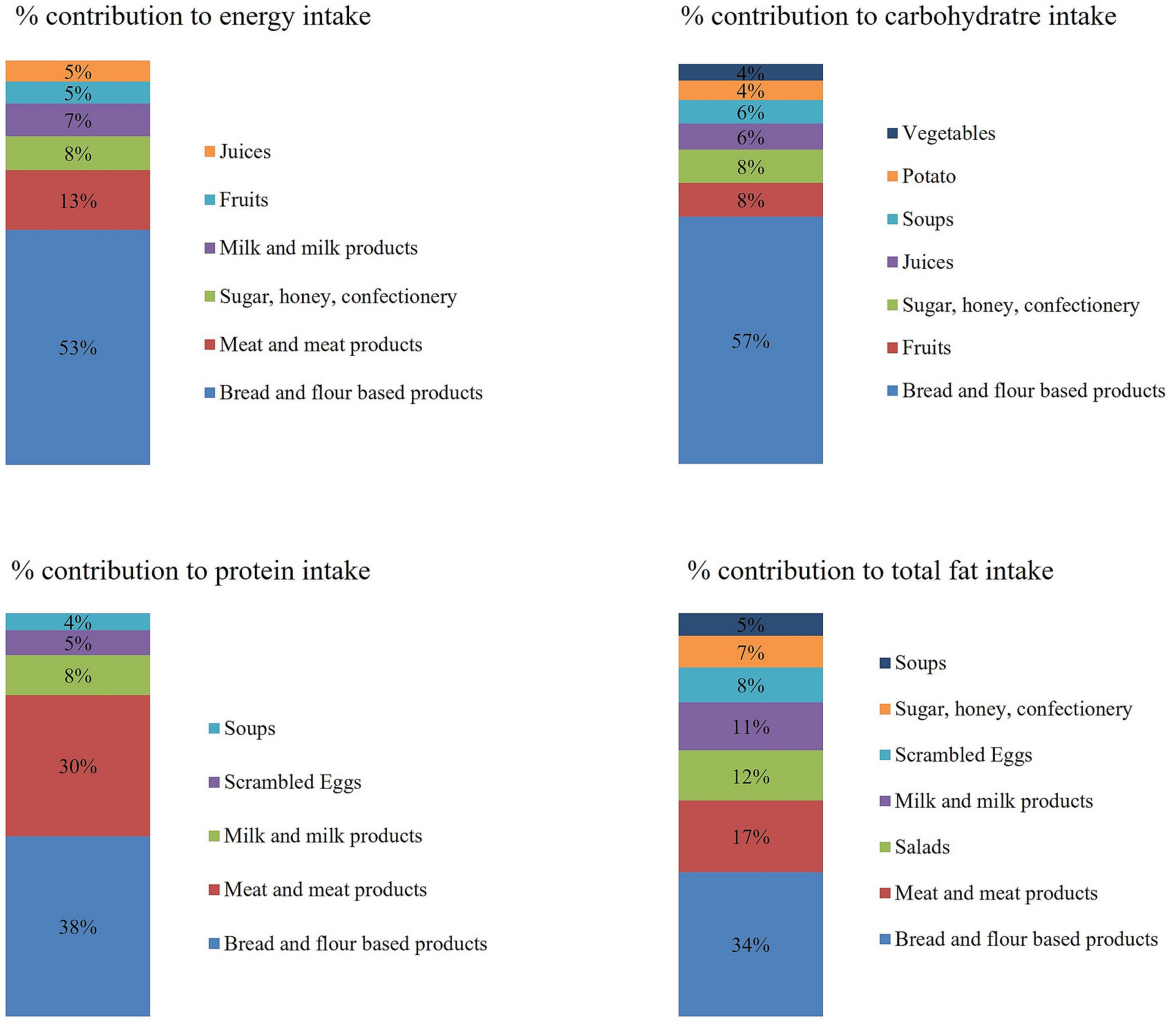
The main contribution of consumed food to total energy and macronutrients during the past 24 h.

In an optimum diet, energy is obtained from a variety of food sources. The estimated mean, 25th, 50th, and 75th percentile intakes of energy and macronutrients during the past 24 h in the case of all the studied 11 food groups and 4 mixed dishes groups are shown in Tables 2–5. Taking into consideration the number of consumers (i.e., 24HR survey respondents who reported consumption of a given food), the contributions of food groups and the mixed dishes groups to intakes of energy and macronutrients in the total population during the past 24 h are shown in Figure 3.

Considering the number of consumers per each food group, estimated mean energy intake is characterized by the consumption of bread and flour-based products (53%), meat and meat products (13% of total energy), sugar, honey, and confectionery (8% of total energy), and milk and milk products (7% of total energy) (Figure 3). It is noteworthy that 50% of bread and flour-based product consumers receive more than 694 kcal energy (approximately more than 40% of total energy) merely from this food group (Table 2). Similarly, carbohydrate intake is attributable mostly to bread and flour-based products (57%), fruits (8%), sugar, honey, and confectionery (8%), and juices (6%). Mixed dishes contribute by 12%, with soups having 6% of the share and salads having 3% of the share (Figure 3). It is noteworthy that 50% of bread and flour-based product consumers receive more than 110 g of carbohydrates from this food group. This is equal to 25% of the estimated mean metabolized energy received from carbohydrates (Table 3). Taking into consideration the number of consumers of each food item, protein intake is also characterized mainly by bread and flour-based products (38%), followed by meat and meat products (30%), and milk and milk products (8%) (Figure 3). Mixed dishes have a contribution of 13% of which scrambled eggs make 5% and beef-containing soups make 4% of protein intake. When considering the 75th percentile intake of bread and bread products and the mean body weight of respondents, the estimated protein intake is equal to 0.55 g/kg, which is slightly less than the AR of 0.63 g/kg (Table 4). Lastly, total fats are received mainly from bread and flour-based products (34%), and meat and meat products (17%). Mixed dishes make up 25% of total fat intake, with salads contributing 12%, scrambled eggs 8%, and soups 5% (Figure 3). The majority of bread and flour-based consumers (75^th^ percentile) receive up to 43.842 g of total fat from this food group (Table 5). Thus, the estimated metabolized energy received from total fats is up to 23E%, coming from the consumption of bread and flour-based products only. Although this number lies between the acceptable RI ranges (20E% - 35E%), it is quite high considering that other widely consumed food items also contain high contents of total fat.

**Table 2:** Energy intake (kcal) received from each food group during the past 24 h.

Food groups	Number of consumers*	Energy intake (kcal)			
		Mean	25%	50%	75%
Bread and flour-based products	1,230	831.014	370.500	694.358	1156.876
Meat and meat products	704	369.943	159.366	305.000	467.518
Fish	28	410.559	351.225	401.400	479.450
Milk and milk products	836	170.113	86.400	137.210	233.640
Eggs	142	133.023	89.100	89.100	178.200
Fat and oil products	132	108.441	65.880	87.840	131.760
Fruits	609	158.439	66.988	116.375	202.624
Vegetables	705	87.368	22.739	48.700	93.301
Potato	404	140.427	142.000	142.000	156.200
Sugar, honey, confectionery	865	169.821	60.000	129.840	223.070
Juices	404	223.898	107.800	200.000	269.871
Salads	392	332.581	264.813	313.950	327.206
Soups	298	262.936	144.000	237.300	371.700
Pilafs	33	400.477	408.000	412.500	417.000
Scrambled eggs	238	388.531	165.500	383.100	537.500

*This indicates the number of respondents out of the studied population (*n* = 1400) who reported to consume that food item or the food in the food group.

**Table 3:** Carbohydrate intake (g) received from each food group during the past 24 h.

Food groups	Number of consumers*	Carbohydrate intake (g)			
		Mean	25%	50%	75%
Bread and flour-based products	1,230	122.999	61.987	110.305	169.870
Meat and meat products	704	5.200	0.000	0.000	2.300
Fish	28	0.000	0.000	0.000	0.000
Milk and milk products	836	5.826	0.640	2.240	10.332
Eggs	142	0.411	0.275	0.275	0.550
Fat and oil products	132	0.000	0.000	0.000	0.000
Fruits	609	33.897	14.480	25.480	42.994
Vegetables	705	14.276	3.578	7.200	14.488
Potato	404	29.074	29.400	29.400	32.340
Sugar, honey, confectionery	865	23.419	8.000	20.844	30.711
Juices	404	39.375	2.800	27.100	58.000
Salads	392	17.710	14.350	14.350	19.363
Soups	298	48.812	13.200	18.625	33.000
Pilafs	33	53.255	35.400	53.100	70.500
Scrambled eggs	238	6.289	1.750	6.900	7.250

*This indicates the number of respondents out of the studied population (*n* = 1400) who reported to consume that food item or the food in the food group.

**Table 4:** Protein intake (g) received from each food group during the past 24 h.

Food groups	Number of consumers*	Protein intake (g)			
		Mean	25%	50%	75%
Bread and flour-based products	1,230	28.205	13.000	26.000	39.000
Meat and meat products	704	38.580	14.700	29.640	52.056
Fish	28	78.246	66.938	76.500	91.375
Milk and milk products	836	9.125	5.120	7.680	12.430
Eggs	142	11.496	7.700	7.700	15.400
Fat and oil products	132	0.000	0.000	0.000	0.000
Fruits	609	2.449	0.825	1.664	3.152
Vegetables	705	3.363	0.950	1.942	3.899
Potato	404	3.362	3.400	3.400	3.740
Sugar, honey, confectionery	865	2.128	0.045	0.993	3.150
Juices	404	0.704	0.245	0.500	0.902
Salads	392	6.767	2.800	2.800	6.850
Soups	298	10.843	5.700	10.671	11.700
Pilafs	33	17.239	12.300	18.450	24.600
Scrambled eggs	238	20.398	6.750	18.600	29.750

*This indicates the number of respondents out of the studied population (*n* = 1400) who reported to consume that food item or the food in the food group.

**Table 5:** Total fat intake (g) received from each food group.

Food Groups	Number of consumers*	Total fats intake (g)			
		Mean	25%	50%	75%
Bread and flour-based products	1,230	24.532	4.250	10.200	43.842
Meat and meat products	704	21.284	7.800	14.560	30.128
Fish	28	10.862	9.293	10.620	12.685
Milk and milk products	836	12.130	6.900	10.080	17.031
Eggs	142	9.443	6.325	6.325	12.650
Fat and oil products	132	12.044	7.317	9.756	14.634
Fruits	609	0.754	0.307	0.500	0.872
Vegetables	705	1.615	0.276	0.607	1.179
Potato	404	0.593	0.600	0.600	0.660
Sugar, honey, confectionery	865	7.695	0.000	4.650	9.938
Juices	404	0.182	0.000	0.245	0.279
Salads	392	26.600	14.000	28.000	28.000
Soups	298	15.914	6.000	14.229	30.000
Pilafs	33	13.616	9.900	14.550	19.200
Scrambled eggs	238	30.231	12.000	30.600	43.250

*This indicates the number of respondents out of the studied population (*n* = 1400) who reported to consume that food item or the food in the food group.

The contributions of food groups to total energy and macronutrient intakes obtained in this study are similar to the 2022 investigation on food consumption and nutrient intakes based on a household budget survey (15). In both studies, bread, and flour-based products account for the majority of energy and macronutrient intakes. Since the studies vary in the amount of investigated food items (164 food items in 24HR and 88 food items in the household budget survey), slight differences are observed in the case of several food groups. Some of these differences can be attributed to the fact that certain ready-to-eat food items (such as pizza, sandwiches, etc.), as well as mixed dishes (such as salads, soups, pilafs, and scrambled eggs), are accounted for in the current 24HR study, but not in the household budget survey.

It is noteworthy, that in developed countries ‘bread and flour-based products’, and ‘sugar and confectionery’ are not always the largest contributors to total energy and macronutrient intake. However, when considering neighbouring countries in the South Caucasus (Georgia, Azerbaijan), it is evident that diets are mainly characterized by staple foods, such as bread and flour-based products. Thus, households can afford staple foods but not nutrient-dense foods. Fruits, vegetables, meats, and dairy products are often replaced with cereals, sweets, and added fats, thus reducing overall dietary nutrient density (14, 33, 34). A comparison with another neighbouring country Iran, further illustrates regional similarities and differences in dietary patterns (35). Although both Armenian and Iranian adult populations experience deviations from dietary recommendations, the nature of these deviations differs. In Iran, the main concern relates to dietary imbalance, particularly excessive fat intake despite relatively adequate macronutrient distribution. In contrast, the present study findings indicate a dual burden characterised by lower energy intake and limited dietary diversity, with a strong dependence on a narrow range of staple foods in Armenia. These peculiarities highlight the importance of country-specific nutritional strategies that address not only nutrient adequacy but also overall dietary structure and diversity.

The observed dietary patterns suggest that macronutrient intake in Armenia is strongly shaped not only by nutritional availability but also by broader structural and behavioural determinants of food choice. A review on of agrifood systems and malnutrition in Armenia reported that a combination of poverty, structural inequalities, and limited labor market opportunities constrains assess to healthy diets, further compounded by low awareness of appropriate dietary practices. The underlying drivers of food insecurity and malnutrition are closely linked o educational level, individual and household dietary behaviors and insufficient awareness of healthy lifestyle in Armenia (36). The observed macronutrient and energy intake patterns in the present study are consistent with this framework, where energy needs are primarily met through inexpensive, carbohydrate-dense foods, while consumption of more nutrient-dense food groups remains limited.

## 4 Conclusion

This study provides a nationally representative assessment of dietary patterns and macronutrient intake among the Armenian adult population based on individual-level data. The study findings indicate that mean energy values for almost all age and gender groups fall below the recommended levels set by EAEU, WHO/FAO, and EFSA. Similarly, average intakes of all macronutrients are generally below EAEU recommended values. When compared with DRVs set by EFSA, only 21% of 24HR survey respondents meet the RI range for carbohydrates, 70% meet the AR for protein, and 25.3% meet the RI range for total fats. A notable finding is the limited dietary diversity observed in the studied population. Armenians’ diet are high in flour-based products, dairy products, sugar, and fat, but low in fish, meat, and eggs. More than 50% of total energy, and carbohydrate intake is attributable to bread and flour-based products, which is indicative of poor dietary diversity. The same food groups are also the largest contributor to protein and total fat intakes. This pattern reflects a reliance on a narrow range of food groups and indicates suboptimal dietary balance.

A key limitation of this study is the use of a single 24HR, which may not fully capture habitual dietary intake and limits the ability to estimate long-term energy and nutrient intake. Hence, future research should incorporate repeated dietary assessments to improve accuracy. Despite this limitation, the study provides the first nationally representative assessment of dietary patterns in Armenia based on individual-level food consumption data. This is a significant strength, as previous evidence has relied on household-level consumption data, which do not allow assessment of individual variability in nutrient intake. The present findings therefore provide a more precise characterization of dietary patterns and macronutrient intake distributions within the population.

Overall, the results highlight the need for targeted public health interventions aimed at improving dietary quality and diversity in Armenia. Priority actions include the development and implementation of national dietary guidelines, strengthening nutrition education programs, and promoting policies that improve access to diverse and nutrient-dense foods. Such measures are essential to address current dietary imbalances, support population health, and reduce the burden of nutrition-related non-communicable diseases in the population.

## Data Availability

The original contributions presented in the study are included in the article/Supplementary material, further inquiries can be directed to the corresponding author/s.
