# Supplementary material for: Assessment of dietary patterns and macronutrient intake among the adult population of Armenia
Source: Front Nutr. 2026 May 20;13:1840868. doi: 10.3389/fnut.2026.1840868 (PMC13229743; doi:10.3389/fnut.2026.1840868)
Supplement: Supplementary file 1 [file Table_1.docx]

**Supplementary Material**

**Table S1.** Food groups (including individual food items and mixed dishes) from 24HR survey

| **Bread and flour-based products** | First-grade wheat bread |
| --- | --- |
|  | Lavash |
|  | Other groats' bread |
|  | Macaroni pasta |
|  | Vermicelli |
|  | Spaghetti |
|  | Other macaronis, pasta |
|  | Biscuits, cakes |
|  | Sour puff pastry (khachapuri with cheese, meat, etc.) |
|  | Sour cakes (tarts with vegetables, cheeses, meat, fish, etc.) |
|  | Crackers, dried bread |
|  | Pizza |
|  | Lahmajo |
|  | Various pastry (waffles, cookies, gingerbread) |
|  | Sandwich, shawarma |
|  | Muesli, oat, corn flakes |
|  | Instant noodles (rolton, doshirak etc.) |
|  | Semolina |
|  | Rice |
|  | Emmer |
|  | Groat |
|  | Buckwheat |
|  | Oat |
|  | Corn (pop-corn) |
| **Milk and milk products** | Pasteurized or sterilized milk |
|  | Condensed, milk including with sugar and cocoa |
|  | Matsun |
|  | Tan |
|  | Sour cream |
|  | Yogurt containing sugar, cocoa or fruits |
|  | Kefir |
|  | Cheese of cow milk |
|  | Curds (cottage cheese) |
| **Meat and meat products** | Beef and veal meat |
|  | Pork meat |
|  | Chicken meat |
|  | Other subproducts (khash) |
|  | Boiled sausage |
|  | Smoked, semi-smoked sausage |
|  | Basturma, soujoukh |
|  | Ham, salted lard |
|  | Sausages/Sausage Wiener |
|  | Other meat products (dried, salted or smoked) |
|  | Other meat products (gavurma) |
|  | Meat cutlet |
|  | Pelmeni, khinkali |
|  | Kebab |
|  | Dolma |
|  | Qyufta |
|  | Other semi-finished products of meat and poultry |
| **Fat and oil products** | Dairy butter, spread |
| **Fish (including canned and smoked products)** | Fresh or chilled fish |
|  | Trout |
|  | Other fishes |
|  | Crawfish |
|  | Other sea products |
|  | Dry, salted or smoked fish |
|  | Dry, salted or smoked other sea products |
|  | Fish or sea product canned |
|  | Fish or sea product caviar |
|  | Otherwise prepare fish or sea product |
| **Eggs (boiled)** | Chicken eggs |
| **Fruits (fresh and processed)** | Apple |
|  | Pear |
|  | Pomegranate |
|  | Apricot |
|  | Peach |
|  | Plum |
|  | Cherry |
|  | Grapes |
|  | Banana |
|  | Fig |
|  | Persimmon |
|  | Orange |
|  | Grapefruit |
|  | Mandarin |
|  | Kiwi |
|  | Lemon |
|  | Watermelon |
|  | Melon |
|  | Strawberry |
|  | Other dried fruits |
| **Vegetables (fresh and processed)** | Cabbage |
|  | Cauliflower |
|  | Broccoli |
|  | Dill, watercress and other mixed herbs |
|  | Tomatoes |
|  | Cucumbers |
|  | Eggplants |
|  | Green and red pepper |
|  | Green beans |
|  | Marrow/Squash |
|  | Carrot |
|  | Onion |
|  | Radish |
|  | Maize |
|  | Mushroom varieties |
|  | Kidney bean |
|  | Lentil |
|  | Tomato paste |
|  | Tomato-containing sauce (including ketchup) |
|  | Preserved olive |
|  | Preserved green peas |
|  | Pickled vegetables |
|  | Caviar (Armenian vegetable mix) |
|  | Grilled vegetables |
| **Potatoes** | Potato (boiled, smashed) |
|  | Potato (fried) |
|  | Chips |
| **Sugar, honey and confectionery** | Sugar |
|  | Apricot and other fruits jam |
|  | Sweet fruit preserve, jam |
|  | Sweet sujukh |
|  | Honey |
|  | Chocolate |
|  | Fruit caramel (local) |
|  | Zefir and sugar confectionery |
|  | Halva |
|  | Ice cream |
| **Juices** | Lemonades, carbonated |
|  | Natural fruit juices |
|  | Vegetable juices |
|  | Fruit and berry compotes |
|  | Other juices/fruit juices with energy |
| **Salads** | Summer salad |
|  | Tabule salad |
|  | Olivier salad |
|  | Cabbage salad |
|  | Salad with chicken meat |
|  | Ceasar salad |
|  | Vinaigrette salad |
|  | Broccoli salad |
|  | Lettuce salad |
|  | Aveluk salad |
|  | Greek salad |
|  | Potato salad |
|  | Salad with crawfish sticks |
|  | Slavonic salad |
|  | Eggplant salad |
|  | Salad with beef |
| **Soups** | Chicken soup |
|  | Beef soup |
|  | Borstch |
|  | Spas |
|  | Lentil soup |
|  | Peas soup |
|  | Bean soup |
|  | Meatball soup |
|  | Mushroom soup |
|  | Summer soup with vegetables |
|  | Broccoli soup |
|  | Soup with rice |
| **Pilafs** | Lentil with rice |
|  | Rice with chicken meat |
|  | Emmer pilaf |
|  | Buckwheat pilaf |
| **Scrambled eggs** | Scrambled eggs |
|  | Scrambled eggs with tomato |
|  | Scrambled eggs with sausages |
|  | Scrambled eggs with cheese |
|  | Scrambled eggs with peas |
|  | Scrambled eggs with green bean |
|  | Scrambled eggs with cauliflower |
|  | Scrambled eggs with greens |
